# Pharmacological LRRK2 kinase inhibition induces LRRK2 protein destabilization and proteasomal degradation

**DOI:** 10.1038/srep33897

**Published:** 2016-09-23

**Authors:** E. Lobbestael, L. Civiero, T. De Wit, J.-M. Taymans, E. Greggio, V. Baekelandt

**Affiliations:** 1 Laboratory for Neurobiology and Gene Therapy, Department of Neurosciences, KU Leuven, Kapucijnenvoer 33, 3000 Leuven, Belgium; 2 Department of Biology, University of Padova, 35131 Padova, Italy; 3 UMR-S1172 Jean-Pierre Aubert Research Center – (INSERM - CHRU de Lille - Université de Lille), Early Stages of Parkinson’s Disease Team, Lille, France

## Abstract

Leucine-rich repeat kinase 2 (LRRK2) kinase activity is increased in several pathogenic mutations, including the most common mutation, G2019S, and is known to play a role in Parkinson’s disease (PD) pathobiology. This has stimulated the development of potent, selective LRRK2 kinase inhibitors as one of the most prevailing disease-modifying therapeutic PD strategies. Although several lines of evidence support beneficial effects of LRRK2 kinase inhibitors, many questions need to be answered before clinical applications can be envisaged. Using six different LRRK2 kinase inhibitors, we show that LRRK2 kinase inhibition induces LRRK2 dephosphorylation and can reduce LRRK2 protein levels of overexpressed wild type and G2019S, but not A2016T or K1906M, LRRK2 as well as endogenous LRRK2 in mouse brain, lung and kidney. The inhibitor-induced reduction in LRRK2 levels could be reversed by proteasomal inhibition, but not by lysosomal inhibition, while mRNA levels remained unaffected. In addition, using LRRK2 S910A and S935A phosphorylation mutants, we show that dephosphorylation of these sites is not required for LRRK2 degradation. Increasing our insight in the molecular and cellular consequences of LRRK2 kinase inhibition will be crucial in the further development of LRRK2-based PD therapies.

Leucine-rich repeat kinase 2 (LRRK2) kinase inhibition is currently one of the prevailing disease-modifying therapeutic strategies for Parkinson’s disease (PD)^1^. LRRK2 is a very attractive target since pathogenic LRRK2 mutations are a common cause of inherited forms of PD^2^,^3^ and genetic variations in the LRRK2 locus are associated with an increased risk to develop sporadic PD^4^,^5^,^6^,^7^. The most common pathogenic mutation, G2019S, increases kinase activity^8^,^9^,^10^, which plays a crucial role in mutant LRRK2-induced toxicity^11^,^12^,^13^ and can be reversed by LRRK2 kinase inhibition^10^,^12^,^14^,^15^. This has stimulated academic and industrial efforts on the development of potent and selective LRRK2 kinase inhibitors^16^,^17^.

LRRK2 is phosphorylated at multiple serines including S910, S935, S955 and S973^18^,^19^,^20^. Although these sites are most likely phosphorylated by other kinases^18^,^19^,^20^,^21^,^22^,^23^,^24^,^25^,^26^,^27^, the LRRK2 kinase domain appears to play a regulatory role in this phosphorylation event since all LRRK2 kinase inhibitors also induce LRRK2 dephosphorylation at S935^22^,^28^,^29^,^30^. Therefore, LRRK2 dephosphorylation at S935 is widely used as a surrogate readout for LRRK2 kinase inhibition in a cellular context^29^,^30^,^31^,^32^.

Before clinical applications can be envisaged, more insight in the molecular and cellular consequences of LRRK2 kinase inhibition will be needed since there might be (side) effects we do not fully understand to date. We have previously shown that LRRK2 kinase inhibition induces PP1-mediated LRRK2 dephosphorylation^33^. Although not proven to be a pathogenic mechanism, the fact that PP1-mediated dephosphorylation is also observed in most pathogenic mutants^19^,^22^,^33^,^34^, calls for caution when considering LRRK2 kinase inhibitors in the clinic. In addition, LRRK2 kinase inhibition can induce LRRK2 ubiquitination^35^ and a reduction of protein levels^35^,^36^,^37^,^38^, which may explain the cellular changes in the lung of non-human primates^36^ or mice^39^ treated with LRRK2 kinase inhibitor, given the close resemblance with the lung phenotype observed in LRRK2 knock-out animals^36^,^37^,^40^,^41^. One of the important outstanding questions is whether this reduction of LRRK2 protein level is purely an unwanted effect or whether this might (partially) account for the beneficial effects of LRRK2 kinase inhibition. LRRK2 ablation was shown to protect against α-synuclein- and LPS-induced toxicity^42^,^43^ and a recent study postulated that reduced LRRK2 protein levels, rather than kinase inhibition, explains the beneficial effects on LRRK2-induced toxicity^38^. Together, these findings underline the importance of understanding LRRK2 kinase inhibitor-induced dephosphorylation and destabilization as a crucial step in the development of LRRK2 kinase inhibition as a PD therapy.

Given the fast LRRK2 dephosphorylation after LRRK2 kinase inhibition^29^, most published reports use kinase inhibitor treatment for a short period of time (minutes to hours). Here, we aimed to assess the effects of sustained LRRK2 kinase inhibition (hours to days) on cellular LRRK2 phosphorylation and protein stability as well as their relationship using phosphorylation mutants. With a view on therapeutic applications, we examined inhibition effects in neuronal and non-neuronal cells using wild type (WT) and pathogenic LRRK2 and different LRRK2 kinase inhibitors.

## Results and Discussion

### Pharmacological LRRK2 kinase inhibition reduces LRRK2 protein levels in overexpressing cells

**Figure 1:**
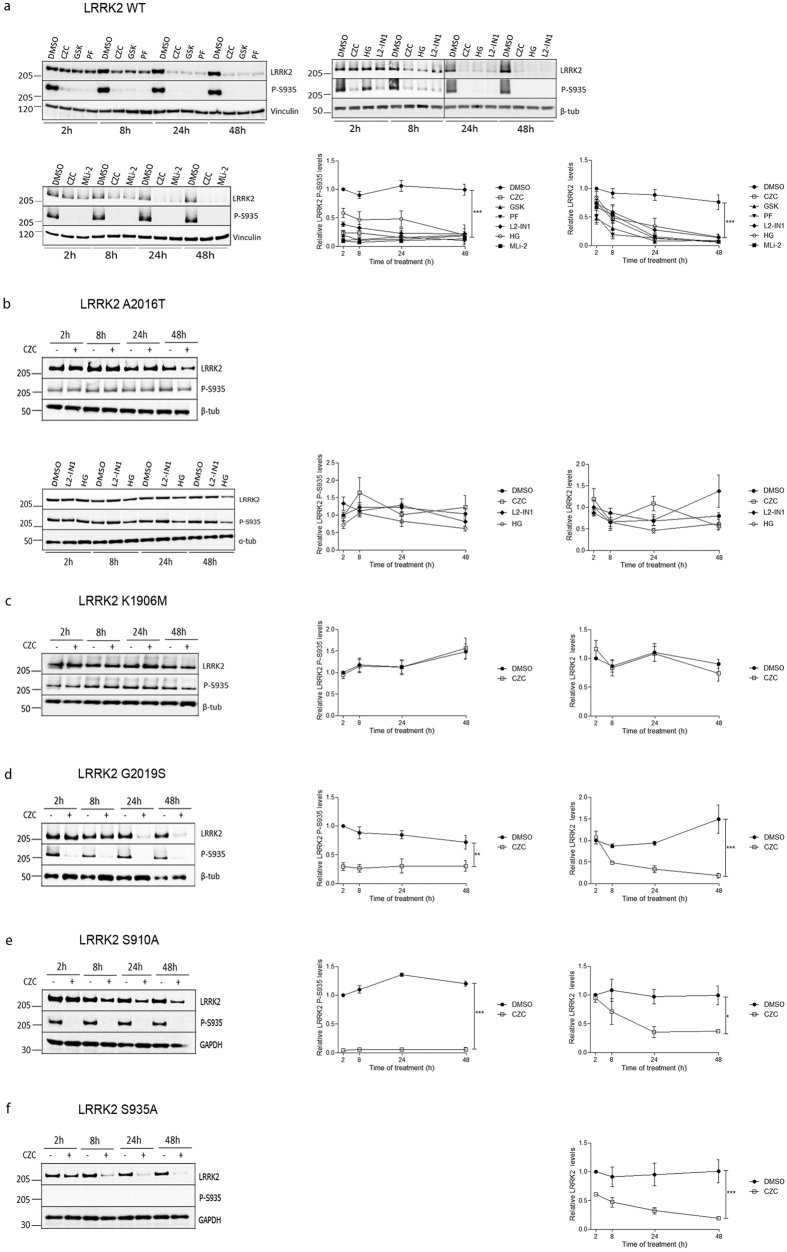
LRRK2 kinase inhibition reduces LRRK2 protein levels. SH-SY5Y overexpressing 3flag-LRRK2 WT (**a**) A2016T (**b**) K1906M (**c**) G2019S (**d**) S910A (**e**) or S935A (**f**) were treated according to different time schedules with LRRK2-IN1 (L2-IN1, 1 μM), CZC-25146 (CZC, 200 nM), PF-06447475 (PF, 150 nM), GSK2578215A (GSK, 1 μM), MLi-2 (10 nM) or HG 10-102-01 (HG, 1 μM) or DMSO. Cell lysates were analyzed with immunoblotting using FlagM2 antibody for LRRK2 detection, anti-LRRK2 P-S935 and anti-α- or β-tubulin, anti-GAPDH or anti-vinculin for equal loading. Shown are representative blots. Graphs show the quantification of blots representing the ratio of phosphorylation at S935 over total LRRK2 signal or total LRRK2 over housekeeping protein signal. Error bars indicate s.e.m. with N ≥ 3. Statistical significance was tested using a two-way ANOVA test with Bonferroni post-tests. ***p < 0.001, **p < 0.01, *p < 0.05.

To investigate the effects of pharmacological LRRK2 kinase inhibition on LRRK2, SH-SY5Y cells with stable lentiviral vector-mediated overexpression of LRRK2 were treated with six different LRRK2 kinase inhibitors: MLi-2^39^, PF-06447475^44^, GSK2578215A^45^, LRRK2-IN1^46^, HG 10-102-01^28^ and CZC-25146^47^ (for more information see^1^). As expected, treatment of cells induced a rapid dephosphorylation at S935. In addition, treatment with each of the inhibitors resulted in a gradual decrease in LRRK2 protein levels, starting from 8 h of treatment (Fig. 1a).

The observation that six different LRRK2 kinase inhibitors, each with different selectivity profiles, reduce LRRK2 protein levels, points to a LRRK2-specific effect. This was further corroborated by the lack of LRRK2 dephosphorylation and protein reduction after treatment of the inhibitor insensitive mutant A2016T^46^,^48^,^49^,^50^ with LRRK2-IN1, CZC-25146 or HG 10-102-01 (Fig. 1b). Interestingly, a trend towards LRRK2 protein destabilization was observed for the HG 10-102-01 compound, which is reported to show strongly reduced activity against LRRK2 A2016T^51^. In addition, also the kinase dead LRRK2 variant K1906M, which was reported to display reduced sensitivity to several LRRK2 kinase inhibitors^24^, did not display S935 dephosphorylation and destabilization upon inhibitor treatment (Fig. 1c).

To examine whether the inhibitor-induced destabilization also holds true for the most common pathogenic mutation G2019S, we performed the same experiment in SH-SY5Y cells stably overexpressing LRRK2 G2019S. As for LRRK2 WT, LRRK2 S935 dephosphorylation was observed starting from the 2 h time point, while LRRK2 levels were decreased from 8 h treatment on (Fig. 1d).

Our results show that LRRK2 kinase inhibition induces LRRK2 dephosphorylation at S935 prior to a reduction in LRRK2 levels. To examine whether dephosphorylation of the cellular LRRK2 phosphorylation sites S910 and S935 is crucial for the reduction of LRRK2 levels, we treated SH-SY5Y cells with stable overexpression of the LRRK2 phosphorylation mutants S910A or S935A (Fig. 1e,f). Interestingly, inhibitor-induced destabilization also occurred in these mutants that cannot be further dephosphorylated at S910 and S935, which indicates that LRRK2 dephosphorylation at S910 and S935 is not required for the reduction of LRRK2 levels. This is in line with the findings of Zhao *et al*.^35^ showing increased ubiquitination of the double S910A/S935A mutant upon inhibitor treatment. Future studies will need to elucidate whether LRRK2 dephosphorylation at other (auto)phosphorylation sites induces the reduction in protein levels or whether other factors such as conformational changes induced by binding of the inhibitor or the LRRK2 kinase inhibition itself, regulate LRRK2 protein levels.

### Reduced LRRK2 protein levels result from increased proteasomal protein degradation

**Figure 2:**
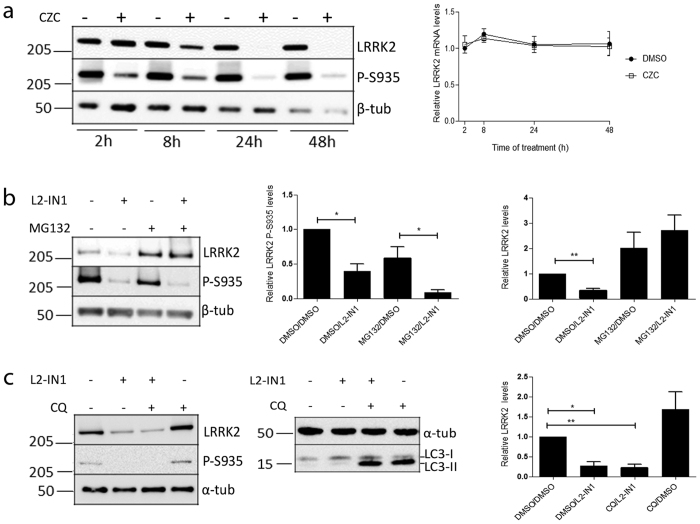
LRRK2 kinase inhibitor-induced reduction of LRRK2 protein levels is caused by proteasomal degradation. (**a**) QPCR analysis was performed on mRNA derived from SH-SY5Y cells overexpressing 3flag-LRRK2 WT, treated with CZC-25146 (CZC, 200 nM) or DMSO for different periods of time. Immunoblotting analysis was performed on cells cultured and treated in parallel with cells for QPCR analysis, using FlagM2 antibody for LRRK2 detection, anti-LRRK2 P-S935 and anti-β-tubulin for equal loading. Shown are representative blots. Quantification of LRRK2 mRNA shows LRRK2 mRNA levels normalized to β-actin mRNA levels with error bars indicating s.e.m., N = 3. (**b**) SH-SY5Y cells overexpressing 3flag-LRRK2 WT were treated for 17 h with LRRK2-IN1 (L2-IN1, 1 μM) and/or MG132 (1 μM), DMSO was used as negative control. Cell lysates were analyzed with immunoblotting using FlagM2 antibody for LRRK2 detection, anti-LRRK2 P-S935 and anti-β-tubulin for equal loading, N ≥ 4. Statistical significance was assessed by column statistics (one-sample t-test) or a nonparametric t-test (Mann-Whitney). **p < 0.01; *p < 0.05. (**c**) SH-SY5Y cells overexpressing 3flag-LRRK2 WT were treated for 48 h with LRRK2-IN1 (L2-IN1, 1 μM) and/or chloroquine (CQ) (10 μM), DMSO was used as negative control. Cell lysates were analyzed with immunoblotting using FlagM2 antibody for LRRK2 detection, anti-LRRK2 P-S935, anti-LC3 antibody and anti-α tubulin for equal loading, N ≥ 4. Statistical significance was assessed by column statistics (one-sample t-test) with Bonferroni correction or a nonparametric t-test (Mann-Whitney). **p < 0.01; *p < 0.05.

Next, we set out to gain mechanistic insight in the LRRK2 kinase inhibitor-induced reduction of LRRK2 protein levels. SH-SY5Y cells, overexpressing LRRK2, were treated for different periods of time with CZC-25146 followed by total RNA isolation and QPCR analysis. In parallel, cells were treated with inhibitor for subsequent immunoblotting analysis to confirm LRRK2 dephosphorylation and protein level reduction. QPCR analysis revealed that, although LRRK2 protein levels were dramatically decreased, LRRK2 mRNA levels remained constant during the entire experiment (Fig. 2a), pointing to a regulation at the protein level rather than a transcriptional event. Since WT LRRK2 has been mainly reported to be degraded by the ubiquitin proteasome system^52^,^53^,^54^,^55^, we treated LRRK2 overexpressing cells with LRRK2 kinase inhibitor together with the proteasomal inhibitor, MG132. Although LRRK2 kinase inhibition still induced LRRK2 dephosphorylation, no decrease in LRRK2 protein levels was observed when proteasomal activity was blocked (Fig. 2b). Conversely, chloroquine-mediated lysosomal inhibition was not able to rescue the LRRK2 kinase inhibitor-induced LRRK2 protein destabilization (Fig. 2c). Together, these findings strongly suggest that the inhibitor-induced reduction of LRRK2 protein levels can be mainly explained by proteasome-mediated LRRK2 degradation. This is in line with a recent report showing increased LRRK2 ubiquitination and degradation after LRRK2 kinase inhibition^35^.

### LRRK2 kinase inhibition reduces endogenous LRRK2 protein levels *in vivo*

**Figure 3:**
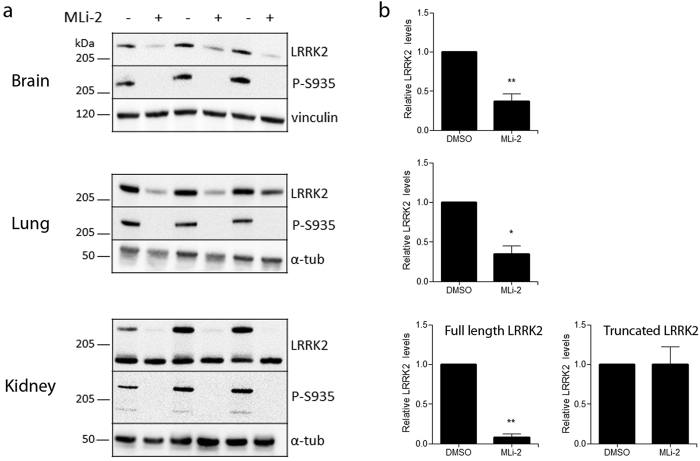
LRRK2 kinase inhibition *in vivo* induces LRRK2 protein destabilization in brain, lung and kidney. C57BL/6J mice received four intraperitoneal injections with 10 mg/kg MLi-2 or DMSO over 30 h. Brain, lung and kidney extracts were taken 2 h after the last injection and analyzed with immunoblotting using MJFF-2 anti-LRRK2 antibody, anti-LRRK2 P-S935 and anti-vinculin or α-tubulin for equal loading. Shown are representative blots with each lane representing a separate animal. Quantification of the blots is represented as a histogram and shows the ratio of total LRRK2 over vinculin or α-tubulin signal, error bars indicate s.e.m. with N ≥ 3, statistical significance was assessed using column statistics. **p < 0.01; *p < 0.05.

To further investigate the *in vivo* relevance of our findings, we examined the effect of LRRK2 kinase inhibition on endogenous LRRK2 in mouse. Mice were injected four times with 10 mg/kg of the highly potent and selective LRRK2 kinase inhibitor MLi-2 or with DMSO. A significant decrease in LRRK2 phosphorylation at S935 and total protein levels was observed in mouse brain (Fig. 3). Given the reported abnormalities in LRRK2 KO mice^56^,^57^,^58^,^59^ and in LRRK2 kinase inhibitor-treated animals^39^,^56^, we also analyzed LRRK2 levels in lung and kidney of mice injected with MLi-2. As in brain, a significant decrease in P-S935 as well as total LRRK2 protein levels was observed in lung and kidney (Fig. 3). A reduction of LRRK2 protein levels in lung and kidney of mice was also reported after in-diet dosing with MLi-2, but no effect on LRRK2 levels in mouse cortex could be observed^39^. Still, a reduction in striatal LRRK2 protein levels has been reported in non-human primates treated with GNE-0877^56^. It will be interesting to further investigate whether this discrepancy can be explained by differences between brain regions, or rather to technical aspects such as buffer composition. Interestingly, in contrast to full length LRRK2, protein stability of the truncated form of LRRK2 in kidney (~170 kD) was not affected upon LRRK2 kinase inhibition, although S935 dephosphorylation was evident (Fig. 3). This is in line with the reported reduction in full length, but not the truncated, LRRK2 protein levels in the kidney of mice treated with a Novartis LRRK2 kinase inhibitor^57^.

### LRRK2 kinase inhibition in primary astrocytes

**Figure 4:**
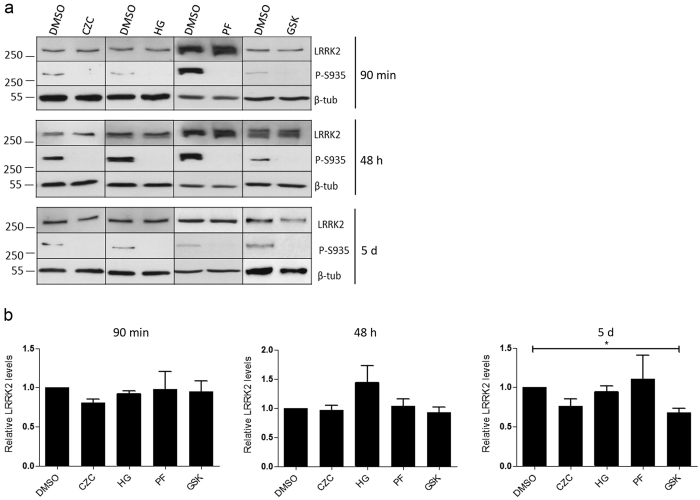
LRRK2 kinase inhibition in primary astrocytes. (**a**) Primary astrocytes were treated with GSK2578215A (GSK, 2 μM), CZC-25146 (CZC, 200 nM), HG 10-102-01 (HG, 1 μM) or PF-06447475 (PF, 150 nM) for 90 min or 48 h or with a tenfold dilution of the concentrations mentioned for 5 or 10 days with fresh compound every two days. Immunoblotting was done with MJFF-2 anti-LRRK2 antibody, anti-LRRK2 P-S935 and anti-β-tubulin for equal loading. Representative blots are shown. (**b**) Quantifications of the blots represent total LRRK2 signal over β-tubulin. Statistical significance was assessed by column statistics (one-sample t-test) with Bonferroni correction for multiple comparisons. *p < 0.05.

To investigate the effect of LRRK2 kinase inhibition on endogenous LRRK2 in brain cells other than neurons, we used primary astrocyte cultures. Treatment of primary astrocytes with the kinase inhibitors GSK2578215A, CZC-25146, HG 10-102-01 or PF-06447475 for up to 48 h did induce significant S935 dephosphorylation but not destabilization of endogenous LRRK2 (Fig. 4). Also sustained LRRK2 kinase inhibition for several days did not induce destabilization, except for the GSK2578215A (Fig. 4).

A more in-depth study in primary astrocytes is needed to explain why not all inhibitors where able to induce LRRK2 destabilization. In addition, it will be interesting to investigate the effects of kinase inhibition in other cell types such as microglial cells.

By using six compounds with different selectivity profiles, the A2016T mutant and different test systems we provide robust evidence that pharmacological LRRK2 kinase inhibition can induce proteasomal LRRK2 protein degradation in different cell types in a LRRK2 inhibitor-specific manner.

This is in line with previous studies reporting reduced LRRK2 protein levels after kinase inhibitor treatment of cells overexpressing LRRK2^35^,^38^, and in kidney^35^,^36^,^37^ or lung^35^ of WT mice. Given the fast effects of LRRK2 kinase inhibition (LRRK2 dephosphorylation within 30 min^29^), most studies have not applied LRRK2 kinase inhibitor treatment for a time span longer than 2 h. This most likely explains why the ‘delayed’ degradation of LRRK2 (more than 2 h after inhibition) in cell lines overexpressing LRRK2 has not been observed in most reports. Interestingly, LRRK2 kinase inhibition *in vivo* does not induce destabilization in all conditions. PF-06447475 treatment in mice for 90 minutes^44^ and in rats for 14 days^60^ does not induce reduced LRRK2 levels in the brain, while we observed a significant reduction of LRRK2 protein levels upon PF-06447475 treatment of LRRK2 overexpressing cell lines. As mentioned before, in-diet MLi-2 treatment of mice caused LRRK2 protein destabilization in lung and kidney, but not in the cortex^39^, while we also observed clear destabilization in full brain extracts. Identification of the key variables in LRRK2 kinase inhibitor-induced LRRK2 protein destabilization will be crucial to explain the observed differences.

Inhibitor-induced LRRK2 protein degradation is an important ‘side’ effect, which needs to be considered in view of clinical applications. Future studies should therefore be directed at understanding how LRRK2 destabilization is triggered and how it takes place. Understanding whether reduced LRRK2 levels are beneficial or rather an unwanted effect of LRRK2 kinase inhibition will be crucial to pursue or redirect current pharmacological LRRK2 therapeutic strategies.

## Materials and Methods

### Antibodies and reagents

LRRK2 kinase inhibitor-1 (L2-IN1), HG 10-102-01 and CZC-25146 were purchased from Calbiochem, GSK2578215A from Tocris and PF-06447475, MG132 and chloroquine from Sigma-Aldrich. MLi-2 was kindly provided by Dr. D. Alessi (Division of Signal Transduction Therapy, University of Dundee). Antibodies used are as follows: anti-flagM2, anti-vinculin and anti-α or β-tubulin (Sigma-Aldrich), anti-LRRK2 MJFF-2, anti-LRRK2 P-S935, anti-LC3 (Novus Biologicals) and anti-GAPDH (Abcam). QPCR probes were from Sigma-Aldrich, QPCR primers from IDT. Lentiviral vectors encoding full length LRRK2 were produced by the Leuven viral vector core (https://gbiomed.kuleuven.be/english/research/50000715/laboratory-of-molecular- virology-and-gene-therapy/lvvc).

### QPCR

LRRK2 mRNA levels were determined using RT-QPCR. Total RNA was reverse transcribed using the High-Capacity cDNA Archive kit (Applied Biosystems). Samples corresponding to 5 μg RNA were subsequently used for QPCR analysis with the iQ5 Multicolor RT-PCR detection system (BioRad). LRRK2 primer/probe set: LRRK2 Fw: 5′-ACGCAGCGAGCATTGTACCTT-3′, LRRK2 Rev: 5′- GGCTTCATGGCATCAACTTCA-3′, LRRK2 probe: 5′-GCTGTCTATGACCTCAGCAAGGGAC AGG C-3′. LRRK2 mRNA levels were normalized to β-actin mRNA levels.

### Cell lines

SH-SY5Y cells stably overexpressing 3flag-LRRK2 (WT or mutants) were generated as described in ref. 30. Cells were maintained in Dulbecco’s modified Eagle’s medium (Gibco-life technologies) supplemented with 15% fetal calf serum (Gibco), 1x non-essential amino acids (Gibco), 200 μg/ml hygromycin and 50 μg/ml gentamycin at 37 °C in a humidified atmosphere containing 5% CO_2_. All cultures were mycoplasma-free. For compound treatment, cells were treated in a 24-well cell culture vessel for the indicated period of time with the compound indicated or DMSO as negative control. Compound concentrations were selected based on the respective cellular IC50 values^1^. To obtain proteasomal or lysosomal inhibition, cells were treated with 1 μM MG132 for 17 h or 10 μM chloroquine for 48 h respectively, DMSO was used as negative control.

For cell lysis, cells were rinsed in PBS and lysed in lysis buffer (Tris 20 mM pH 7.5, NaCl 150 mM, EDTA 1 mM, Triton 1%, glycerol 10%, protease inhibitor cocktail (Roche) and phosphatase inhibitor (PhosStop, Roche)). Cell lysates were cleared by centrifugation at 14,000 g for 10 min and further analyzed via immunoblot.

### Primary cortical astrocyte cultures

Mixed cortical cell isolation for astrocyte cultures were performed using P1 to P4 mouse pups. Isolated cortex was mechanically dissociated and cells were cultured in BME supplemented with 10% FBS. After 10 days in culture, astrocyte purity was examined by immunofluorescence with anti-GFAP antibody (Dako).

### Brain extracts

All animal experiments were performed in accordance with the European Communities Council Directive of November 24, 1986 (86/609/EEC) and approved by the Bioethical Committee of the KU Leuven (Belgium). C57BL/6J mice were injected i.p. with 10 mg/kg MLi-2 or DMSO in hydroxypropyl-β-cyclodextrin (Sigma-Aldrich) and PBS. Four injections were given in total over 30 h and animals were sacrificed 2 h after the last injection. Whole brain, lung and kidney extracts were lysed in sucrose buffer (10 mM TrisHCl, 1 mM EDTA, 0.25 mM sucrose, protease inhibitor cocktail and phosphatase inhibitor) using a dounce homogenizer. Brain extracts were cleared by 10 min centrifugation at 3000 g followed by centrifugation of the supernatant for 30 min at 20000 g.

### Immunoblotting

Protein content of cell lysates was determined using the bicinchoninic acid (BCA) protein determination assay (Pierce Biotechnology). Cell lysates were resolved by electrophoresis on an in-house 12.5% polyacrylamide gel or a NuPage 3–8% tris-acetate gradient gel. Separated proteins were transferred to a polyvinylidene fluoride membrane (Bio-Rad) and non-specific binding sites were blocked for 30 min in PBS with 0.1% Triton X-100 (PBST) and 5% non-fat milk. After overnight incubation at 4 °C with primary antibo-dies, blots were washed 3 times with PBST, incubated with horseradish peroxidase-conjugated secondary antibody (Dako, Glostrup) for 1 h and washed again. Bands were visualized using enhanced chemiluminescence (Amersham Pharmacia Biotech). To normalize the signal of phospho-specific antibodies to LRRK2 expression levels, blots were stripped after detection of the LRRK2 signal and reprobed with anti-phospho-LRRK2 antibody by incubating the blot with stripping buffer (62.5 mM Tris-HCl pH 6.8, 2% SDS and 100 mM β-mercaptoethanol) for 30 min at 70 °C, followed by 2 10 min wash steps with PBST. Densitometric analysis of the bands on the blot autoradiograms was performed with Aida analyzer v1.0 (Raytest).

### Statistics

Figures shown are representative of at least three independent experiments. Phosphorylation levels were normalized for expression levels and experimental test conditions for control conditions. Statistical analysis was performed with a t-test, two-way ANOVA test or column statistics (one-sample t-test) comparing test values to the hypothetical value of 1 with Bonferroni correction for multiple comparisons. Statistical significance was set at p < 0.05.

## Additional Information

**How to cite this article**: Lobbestael, E. *et al*. Pharmacological LRRK2 kinase inhibition induces LRRK2 protein destabilization and proteasomal degradation. *Sci. Rep.* **6**, 33897; doi: 10.1038/srep33897 (2016).
